# Joint Subchannel Pairing and Power Control for Cognitive Radio Networks with Amplify-and-Forward Relaying

**DOI:** 10.1155/2014/380106

**Published:** 2014-06-15

**Authors:** Yanyan Shen, Shuqiang Wang, Zhiming Wei

**Affiliations:** ^1^Department of Mechanical and Biomedical Engineering, City University of Hong Kong, Kowloon, Hong Kong; ^2^Shenzhen Institutes of Advanced Technology, Chinese Academy of Sciences, Shenzhen, Guangdong Province 518000, China; ^3^Shandong Academy of Agricultural Machinery Sciences, Jinan, Shandong Province 250000, China

## Abstract

Dynamic spectrum sharing has drawn intensive attention in cognitive radio networks. The secondary users are allowed to use the available spectrum to transmit data if the interference to the primary users is maintained at a low level. Cooperative transmission for secondary users can reduce the transmission power and thus improve the performance further. We study the joint subchannel pairing and power allocation problem in relay-based cognitive radio networks. The objective is to maximize the sum rate of the secondary user that is helped by an amplify-and-forward relay. The individual power constraints at the source and the relay, the subchannel pairing constraints, and the interference power constraints are considered. The problem under consideration is formulated as a mixed integer programming problem. By the dual decomposition method, a joint optimal subchannel pairing and power allocation algorithm is proposed. To reduce the computational complexity, two suboptimal algorithms are developed. Simulations have been conducted to verify the performance of the proposed algorithms in terms of sum rate and average running time under different conditions.

## 1. Introduction

With the emergence of wireless services and applications, the demand for spectrum is increasing. However, experimental results have demonstrated that traditional fixed spectrum allocation schemes lead to inefficient spectrum utilization [1]. To improve the spectrum utilization, cognitive radio networks (CRNs) that allow the unlicensed secondary users (SUs) to share the licensed spectrum with the licensed primary users (PUs) have been proposed.

Based on whether the PUs are aware of the existence of the SUs, the dynamic spectrum sharing in CRNs can be divided into two categories. For the first category, the PUs are not aware of the existence of the SUs. The SUs are able to detect the available spectrums and then use them for their own data transmission if the interference to the PUs is not harmful. For the second category, the PUs leverage parts of its spectrums to the SUs in exchange for appropriate remuneration.

Recently, by exploiting the cooperative diversity, the cooperation technique has been incorporated into the CRNs [2, 3]. Based on the above categories for the dynamic spectrum sharing, the cooperation in CRNs can be divided into two categories as well [4]. For the first category, the SUs are located in different areas and thus have different channel conditions. In this case, some SUs may be selected as relays to help forward other SUs' data [5–9]. It is named as cooperation among SUs. For the second category, the PUs lease some resources (time or frequency) to the SUs; thus the SUs will act as relays to forward the PUs' data. When the data of the PUs are completely transmitted or the data rate requirements of the PUs are satisfied, then the SUs can transmit their own data [10–12]. We call it cooperation among PUs and SUs. In this paper, we will consider the cooperation among SUs.

The resource allocation problem is significant for CRNs with cooperation among SUs. A typical feature of CRNs, which differs from other kinds of networks, is that the quality of service (QoS) of the PUs should be guaranteed by imposing interference power constraints on the resource allocation problem [13–15]. For the resource allocation problem in relay networks, the problem of power allocation and relay selection has been investigated in [16, 17]. In particular, if wireless relay networks have multiple channels, for example, orthogonal frequency-division multiplexing (OFDM) relay networks, the resource allocation problem including the subchannel allocation and subchannel pairing has also been studied. The existing literature has shown that appropriate subchannel pairing has improved the network performance for not only the amplify-and-forward (AF) relay strategy but also the decode-and-forward (DF) relay strategy [18–22]. However, there have been very few studies devoted to the resource allocation problem with subchannel pairing in relay-based CRNs.

Some studies have investigated the resource allocation problem for the case that secondary relay helps SU's transmissions in CRNs. Resource allocation in the downlink transmission over a cluster-based cellular network was analyzed in [23], where relays were fixed and supposed to be able to help forward data from the base station to the SUs in its cluster. The formulated problem was an average weighted sum throughput maximization problem; then the dual decomposition method was used to derive an asymptotically optimal algorithm. The relay selection and power allocation problem has been investigated in [5, 6]. The problems under consideration in [5, 6] were formulated as optimization problems under the interference power constraint and the total power constraint; however, the network models were different. Li et al. [5] considered one channel in the model, but Bharadia et al. [6] considered multiple channels. Although there were multiple channels, the channel pairing problem was not considered in [6]. Meanwhile, the power constraint in [6] was the total source and relay power constraint. This constraint can give some insight for the power allocation but is not very suitable for modeling the practical networks where the source and the relay usually have their individual power supplies. Hence, individual source and relay power constraints are more appropriate for modeling the wireless relay networks in reality, and we will study the resource allocation under individual source and relay power constraint in this paper. Soury et al. in [7] studied the subchannel pairing, power allocation, and relay strategy selection (AF and DF selection) problem in CRNs. Although this paper considered subchannel pairing, the optimal subchannel pairing solution was not given. Sidhu et al. in [8] studied the joint subchannel pairing and power allocation problem for AF relay-based CRNs. Our paper is related to [8]. However, it should be noted that the network models in [8] and our paper are very different. To make the network model simple, the direct transmission link from the secondary source node to the secondary destination node is missing in [8], while both the direct transmission link and the relay transmission link are considered in our paper. From this point of view, the network model in [8] can be regarded as a special case of our model. Hence, the network model in our paper is more general than that in [8]. In addition, the proposed algorithms in [8] cannot be directly applied to solve the problem here. In Section 5.2, the performance comparison of the proposed algorithms in this paper and the optimal algorithm in [8] will be analyzed.

In this paper, we will investigate the joint subchannel pairing and power allocation problem in CRNs where a SU is helped by a secondary relay with AF relay strategy. Both the direct transmission link and the relay transmission link are considered. Our objective is to maximize the SU's sum rate on all subchannels subject to individual power constraints at the source and the relay, the interference power constraints to the PU, and the subchannel pairing constraint. The problem is formulated as a mixed integer programming problem. We jointly optimize the subchannel pairing and power allocation by the dual decomposition method. To reduce the computational complexity further, two suboptimal algorithms are proposed as well. Simulation results show that the two suboptimal algorithms spend much less running time achieving sum rates that are close to the optimal solution under various simulation conditions.

The remainder of the paper is organized as follows.  Section 2 describes the system model and problem formulation. The optimal solution is proposed in Section 3. Two suboptimal algorithms with lower computational complexity are proposed in Section 4. Performance evaluation of the proposed algorithms is shown in Section 5, and Section 6 concludes the work.

## 2. System Model and Problem Formulation

**Figure 1:**
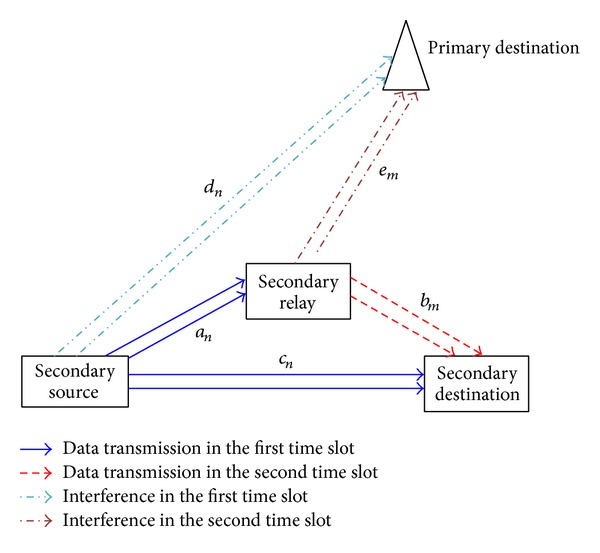
System model.

Consider a network setting where a PU and a SU coexist, which is shown in Figure 1. The SU can adopt the available channels that are licensed to the PU for its own data transmission. It is assumed that the total available bandwidth that is licensed to the PU is *B* Hz. The total bandwidth is divided into *N* nonoverlapping subchannels each with *B*/*N* Hz bandwidth. Let **N** = {1,2,…, *N*} denote the set of subchannels. Since the SU may cause interference to the PU, relay-based transmission is adopted by the SU to reduce power; thus it can ease the interference to the PU. For simplicity, we will use source, relay, and destination to denote secondary source, secondary relay, and secondary destination in the following.

The channel gains of subchannel *n* from the source to the destination and from the source to the relay are denoted by *g*_*s*,*d*_^*n*^ and *g*_*s*,*r*_^*n*^, respectively, and those of subchannel *m* from the relay to the destination are denoted by *g*_*r*,*d*_^*m*^. *σ*_*s*,*d*_^*n*^, *σ*_*s*,*r*_^*n*^, and *σ*_*r*,*d*_^*m*^ are the variances of the additive white Gaussian noises (AWGN) in the corresponding subchannels, respectively. It is assumed that perfect channel state information of each subchannel is known by a central controller. For notational brevity, let *a*_*n*_ = *g*_*s*,*r*_^*n*^/*σ*_*s*,*r*_^*n*^, *b*_*m*_ = *g*_*r*,*d*_^*m*^/*σ*_*r*,*d*_^*m*^, and *c*_*n*_ = *g*_*s*,*d*_^*n*^/*σ*_*s*,*d*_^*n*^. AF relay strategy is used by the relay. It is assumed that the time domain is equally divided into a number of time slots. The AF cooperative transmission is a two-time slot process. In the first time slot, the secondary source uses power *P*_*n*,*m*_^*s*^ to transmit data on subchannel *n*; the secondary relay and destination can receive signal from it. In the second time slot, the relay amplifies the received signal and uses power *P*_*n*,*m*_^*r*^ to forward it to the destination. At the end of the second time slot, the destination combines the received signals from both the relay and the source using the maximum ratio combiner. To improve the transmission rate further, subchannel pairing is considered. Let *ρ*_*n*,*m*_ denote the subchannel pairing in the two time slots. If subchannel *n* in the first time slot and subchannel *m* in the second time slot are paired, *ρ*_*n*,*m*_ = 1; otherwise *ρ*_*n*,*m*_ = 0. It is assumed that the signals transmitted on different subchannel pairings are different. The data rate (bps/Hz) for the cooperative transmission on subchannel pairing (*n*, *m*) is denoted by Li et al. [19]

(1) $$\begin{array}{c} R_{n,m}=\frac{1}{2}\log_{2}\left(1+c_{n}P_{n,m}^{s}+\frac{a_{n}P_{n,m}^{s}b_{m}P_{n,m}^{r}}{1+a_{n}P_{n,m}^{s}+b_{m}P_{n,m}^{r}}\right), \end{array}$$

which is not jointly concave with respect to *P*_*n*,*m*_^*s*^ and *P*_*n*,*m*_^*r*^. To facilitate the analysis in the following, it can be approximated by

(2) $$\begin{array}{c} R_{n,m}=\frac{1}{2}\log_{2}\left(1+c_{n}P_{n,m}^{s}+\frac{a_{n}P_{n,m}^{s}b_{m}P_{n,m}^{r}}{a_{n}P_{n,m}^{s}+b_{m}P_{n,m}^{r}}\right). \end{array}$$

Such approximation has also been used in [19–22], and the approximation gap disappears as the signal-to-noise ratio becomes large.

To protect the PU's QoS, the interference to the primary destination should not be larger than the given threshold *T*_1_ and *T*_2_ in the first and second time slot, respectively, which can be expressed by

(3) $$\begin{array}{c} \overset{N}{\underset{n=1}{\sum }}P_{n,m}^{s}d_{n}\leq T_{1}, \end{array}$$

(4) $$\begin{array}{c} \overset{N}{\underset{m=1}{\sum }}P_{n,m}^{r}e_{m}\leq T_{2}, \end{array}$$

where *d*_*n*_ = *g*_*s*,*p*_^*n*^/*σ*_*s*,*p*_^*n*^ and *e*_*m*_ = *g*_*r*,*p*_^*m*^/*σ*_*r*,*p*_^*m*^ and *g*_*s*,*p*_^*n*^ and *g*_*r*,*p*_^*m*^ are the channel gain from the source to the primary destination on subchannel *n* and that from the relay to the primary destination on subchannel *m*, respectively. *σ*_*s*,*p*_^*n*^ and *σ*_*r*,*p*_^*m*^ are the variances of AWGN on the corresponding subchannels. It should be noted that *T*_1_ and *T*_2_ might have the same value in practice if the interference tolerance level of the PU is a constant or the required QoSs of the PU are stable in a given time period. Here, we use *T*_1_ and *T*_2_ to describe a general relay-based CRN model, and the following results in the paper can be easily extended to the case where *T*_1_ = *T*_2_.

We desire to study the subchannel pairing and power allocation problem to maximize the transmission rate of the SU under the source power constraint, the relay power constraint, the subchannel pairing constraint, and the interference power constraint. The problem under consideration can be formulated as follows:

(5) max⁡ρn,m,Pn,ms,Pn,mr ∑n=1N∑ m=1Nρn,mRn,mC1.  ∑n=1NPn,ms≤PSC2.  ∑m=1NPn,mr≤PRC3.  0⩽Pn,ms, 0⩽Pn,mr, ∀n, ∀ms.t.C4.  ∑n=1Nρn,m=1, ∀mC5.  ∑m=1Nρn,m=1, ∀nC6.  ρn,m∈{0,1}, ∀n, ∀mC7.  (3)  and  (4),

where *C*1 and *C*2 represent the individual source and relay power constraints; *P*_*S*_ and *P*_*R*_ are the total power at the source and the relay, respectively. *C*3 indicates that the consumed power on each subchannel at the source and the relay should be nonnegative. *C*4~*C*6 are the subchannel pairing constraints. *C*6 reveals that the subchannel pairing indicator *ρ*_*n*,*m*_ is a binary variable. Combined with *C*6, *C*4 indicates that, for any subchannel *m* in the second time slot, at most one subchannel in the first time slot can be paired with it, and *C*5 indicates that, for any subchannel *n* in the first time slot, at most one subchannel in the second time slot can be paired with it. *C*7 is the interference power constraints to the primary destination.

## 3. Optimal Solution

Problem (5) is a mixed integer programming problem; it is generally difficult to obtain the optimal solution. Fortunately, when the number of subchannels goes to infinity, the dual gap between the primal problem and the dual problem goes to zero [24]; thus the dual decomposition method can be used to solve this problem.

### 3.1. Dual Problem

By introducing the nonnegative Lagrange multipliers ***λ*** = [*λ*_1_, *λ*_2_, *λ*_3_, *λ*_4_] to the constraints *C*1, *C*2, and *C*7, the partial Lagrange function can be expressed as

(6) L=∑n=1N∑ m=1Nρn,mRn,m+λ1(PS−∑n=1NPn,ms) +λ2(PR−∑m=1NPn,mr)+λ3(T1−∑n=1NPn,msdn) +λ4(T2−∑m=1NPn,mrem).

Thus the dual problem is

(7) min⁡λ g(λ)s.t. λ⩾0,

where *g*(***λ***) is the dual function and is given by

(8) g(λ)=max⁡ρn,m,Pn,ms,Pn,mr L   s.t. C3,C4,C5,  and  C6.

### 3.2. Solution to the Dual Problem

The dual function *g*(***λ***) is not differential due to the discontinuity of the subchannel pairing variable *ρ*_*n*,*m*_, and hence its gradient does not exist. Instead, the subgradient method can be adopted. The subgradient of *g*(***λ***) is

(9) Δλ1=PS−∑n=1Nρ¯n,mP¯n,ms,Δλ2=PR−∑m=1Nρ¯n,mP¯n,mr,Δλ3=T1−∑n=1Nρ¯n,mP¯n,msdn,Δλ4=T2−∑m=1Nρ¯n,mP¯n,mrem,

where ${\bar{\rho }}_{n,m}$, ${\bar{P}}_{n,m}^{s}$, and ${\bar{P}}_{n,m}^{r}$ are the solution to (8). We will discuss how to solve (8) in the following. Based on the subgradient of *g*(***λ***), the update of the dual variable ***λ*** is given by

(10) $$\begin{array}{c} \lambda \left(t+1\right)={\left[\lambda \left(t\right)-\gamma \left(t\right)\Delta \lambda \right]}^{+}, \end{array}$$

where *t* is the iteration number, *γ*(*t*) is the step size at iteration *t*, and [*x*]^+^ = max⁡(*x*, 0). If *γ*(*t*) satisfies the diminishing step size rule, then by using the subgradient method in (10), ***λ*** is guaranteed to converge to the optimal $\bar{\lambda }$ [25].

Next, we will deduce the optimal subchannel pairing and power allocation, which is the solution to problem (8). Through some algebra operations, *L* can be rewritten as

(11) L=∑n=1N∑ m=1Nρn,mRn,m−λ1∑n=1NPn,ms−λ2∑m=1NPn,mr −λ3∑n=1NPn,msdn−λ4∑m=1NPn,mrem+λ1PS +λ2PR+λ3T1+λ4T2.

To facilitate the calculation of the optimal power and subchannel pairing, *λ*_1_ *P*_*S*_ + *λ*_2_ *P*_*R*_ + *λ*_3_ *T*_1_ + *λ*_4_ *T*_2_ in (11) can be deleted without impact on deriving the optimal power allocation, and thus, *L* can be expressed by

(12) $$\begin{array}{c} L=\overset{N}{\underset{n=1}{\sum }}\overset{N}{\underset{\;m=1}{\sum }}\rho_{n,m}U_{n,m}, \end{array}$$

where

(13) Un,m=Rn,m−λ1∑n=1NPn,ms−λ2∑m=1NPn,mr −λ3∑n=1NPn,msdn−λ4∑m=1NPn,mrem.

Taking the first-order derivatives of *L* with respect to *P*_*n*,*m*_^*s*^ and *P*_*n*,*m*_^*r*^ and making them equal 0, then we get

(14) ∂L∂Pn,ms=12ln⁡2((cn(anPn,ms+bmPn,mr)2+anbm2Pn,mr2) ×((1+cnpn)(anPn,ms+bmPn,mr)2   +anPn,msbmPn,mr(anPn,ms+bmPn,mr))−1) −(λ1+λ3dn)=0,

(15) ∂L∂Pn,mr =12ln⁡2((an2bmPn,ms2)     ×((1+cnpn)(anPn,ms+bmPn,mr)2       +anPn,msbmPn,mr(anPn,ms+bmPn,mr))−1)  −(λ2+λ4em)=0.

With (14) and (15), we obtain that at the optimal point

(16) $$\begin{array}{c} {\bar{P}}_{n,m}^{s}=A{\bar{P}}_{n,m}^{r}, \end{array}$$

where

(17) A=bm(λ2+λ4em)cn[(λ1+λ3dn)bm−(λ2+λ4em)cn]an +((bm(((λ2+λ4em)cn)2     +((λ1+λ3dn)bm−(λ2+λ4em)cn)     ×(cn+an)(λ2+λ4em))1/2) ×([(λ1+λ3dn)bm−(λ2+λ4em)cn]an)−1).

To make sure *A*⩾0, (*λ*_1_ + *λ*_3_ *d*_*n*_)*b*_*m*_ − (*λ*_2_ + *λ*_4_ *e*_*m*_)*c*_*n*_⩾0. Substituting (16) into (15), then we get the optimal ${\bar{P}}_{n,m}^{s}$ and ${\bar{P}}_{n,m}^{r}$ shown as

(18) P¯n,mr={Γ,if  (λ1+λ3dn)bm−(λ2+λ4em)cn≥00,else,

(19) P¯n,ms={AP¯n,mr,if  P¯n,mr≠0,Θ,else,

where

(20) $$\begin{array}{c} \Gamma =\frac{\left(1/2\left(\ln2\right)\right){\left(a_{n}A\right)}^{2}b_{m}-\left(\lambda_{2}+\lambda_{4}e_{m}\right){\left(a_{n}A+b_{m}\right)}^{2}}{\left(\lambda_{2}+\lambda_{4}e_{m}\right)\left(c_{n}a_{n}A+c_{n}b_{m}+a_{n}b_{m}\right)\left(a_{n}A+b_{m}\right)A},\\ \Theta ={\left[\frac{1}{2(\ln2)\left(\lambda_{1}+\lambda_{3}d_{n}\right)}-\frac{1}{c_{n}}\right]}^{+}. \end{array}$$

Substituting ${\bar{P}}_{n,m}^{s}$ and ${\bar{P}}_{n,m}^{r}$ into (13), we get

(21) $$\begin{array}{c} L=\overset{N}{\underset{n=1}{\sum }}\overset{N}{\underset{\;m=1}{\sum }}\rho_{n,m}U_{n,m}\left({\bar{P}}_{n,m}^{s},{\bar{P}}_{n,m}^{r}\right). \end{array}$$

Since the aim of (8) is to get the maximal of *L*, intuitively, the subchannel pairing solution can be determined by

(22) $$\begin{array}{c} {\bar{\rho }}_{n,m}=\{\begin{array}{cc} 1, & \text{if}\;\;m=\;\underset{m\in N}{\arg\;\max}\;U_{n,m},\\ 0, & \text{else}. \end{array} \end{array}$$

It should be noted that although (22) can guarantee that the constraint *C*5 holds, it cannot guarantee that the constraint *C*4 simultaneously holds. That is to say, the obtained ${\bar{\rho }}_{n,m}$ from (22) may correspond to a situation where more than one subchannel in the first time slot might be paired with the same subchannel in the second time slot, for example, the subchannel pairing solutions ${\bar{\rho }}_{1,3}=1$ and ${\bar{\rho }}_{2,3}=1$. As a result, the subchannel pairing obtained from (22) may be infeasible. To guarantee the subchannel pairing constraints *C*4, *C*5, and *C*6 are satisfied simultaneously, one naive method is the exhaustive search. However, its computational complexity is *N*!, which is prohibitive from practical implementation, especially when the number of subchannels is large. In order to reduce the computational complexity without loss of optimality, we will employ the Hungarian method to solve the subchannel pairing problem.

Define matrix **U** = [*U*_*n*,*m*_]_*N*×*N*_; then the subchannel pairing problem is equivalent to an assignment problem. The rule is to pick up one and only one entry from each row and each column of **U** to make the sum of the picked up entries maximal. The Hungarian method with the computational complexity *O*(*N* ^3^) can solve this kind of problem efficiently. The Hungarian method for subchannel pairing is described in Algorithm 1. The whole algorithm is shown in Algorithm 2, which is a joint optimal subchannel pairing and power allocation algorithm.

Although Algorithm 2 can get the solution to problem (5), it is observed from the simulation results that the source power constraints *C*1 with (3) and the relay power constraint *C*2 with (4) are hard to be satisfied with equality. Because (5) is a maximization problem with an objective function that is increasing with respect to *P*_*n*,*m*_^*s*^ and *P*_*n*,*m*_^*r*^, thus, at the optimal point, at least one of the source power constraints *C*1 and (3) should be met strictly. And it also holds for the relay power constraints *C*2 and (4). Hence, the power obtained from Algorithm 2 should be scaled until at least one of the source power constraints *C*1 and (3)  and at least one of the relay power constraints *C*2 and (4) are satisfied with equality.

## 4. Suboptimal Solutions

In this section, two suboptimal algorithms with low computational complexity will be proposed.

### 4.1. Suboptimal Algorithm  1

Suboptimal algorithm 1 is a joint fixed subchannel pairing and optimal power allocation algorithm. The subchannel pairing is predetermined; for example, subchannel *n* in the first time slot is paired with subchannel *n* in the second time slot, and thus, ${\bar{\rho }}_{n,n}=1$ and ${\bar{\rho }}_{n,m}=0$, ∀*n*, *m* ∈ **N** and *m* ≠ *n*. When the subchannel pairing is determined, the power allocation problem becomes much simpler than problem (6). And the dual decomposition method used in the previous section can be adopted here. It should especially be pointed that from the subchannel pairings ${\bar{\rho }}_{n,n}=1$ and ${\bar{\rho }}_{n,m}=0$, ∀*n*, *m* ∈ **N** and *m* ≠ *n*, it is known that ${\bar{P}}_{n,m}^{s}=0$ and ${\bar{P}}_{n,m}^{r}=0$, ∀*n*, *m* ∈ **N** and *m* ≠ *n*, and thus only ${\bar{P}}_{n,n}^{s}$ and ${\bar{P}}_{n,n}^{r}$, ∀*n* ∈ **N** need to be calculated in each iteration. The total algorithm is shown in Algorithm 3.

Compared with Algorithm 2, this algorithm has similar iteration process as Algorithm 2 expect for two aspects. First, this algorithm does not need to calculate the subchannel pairing by the Hungarian method, but Algorithm 2 needs this. Second, this algorithm only needs to calculate the power ${\bar{P}}_{n,n}^{s}$ and ${\bar{P}}_{n,n}^{r}$, ∀*n* ∈ **N**, but Algorithm 2 needs to calculate ${\bar{P}}_{n,m}^{s}$ and ${\bar{P}}_{n,m}^{r}$, ∀*n*, *m* ∈ **N**.

As in Algorithm 2, the power obtained by this algorithm cannot make the power constraints *C*1, *C*2, and *C*7 in (5) be satisfied with equality. Therefore, the same power scaling as Algorithm 2 will be used at the end of this algorithm.

### 4.2. Suboptimal Algorithm  2

Suboptimal algorithm  2 is a joint optimal subchannel pairing and equal power allocation algorithm. The optimal subchannel pairing is calculated by the Hungarian method. For equal power allocation, both total power constraints and interference power constrains should be satisfied. This is achieved by first ignoring the interference power constraints and allocating the total power equally on each subchannel and then checking the interference power constraints and conducting some operations until the interference power constraints are satisfied. The iteration process of this algorithm is shown as follows, and the entire suboptimal algorithm  2 is described in Algorithm 4.

**Step 1 .**

Without the consideration of the interference power constraints (3) and (4), the total power is equally distributed to each subchannel; that is, *P*_*n*,*m*_^*s*^ = *P*_*S*_/*N*, *P*_*n*,*m*_^*r*^ = *P*_*R*_/*N*, ∀*n* ∈ **N**, ∀*m* ∈ **N**. Then the Hungarian method is used for the subchannel pairing.

**Step 2 .**

Based on the allocated power and subchannel pairing, the interference power constraints (3) and (4) will be verified. If the constraints (3) and (4) are satisfied, stop the iteration of the algorithm and now the solution is the final solution; otherwise, reduce the total power consumption on all the subchannels. For the reduction of the total power consumption, three cases should be considered. (a) If (3) is violated but (4) is satisfied, then reduce the total source power *P*_*S*_ by a small constant Δ  (Δ > 0). (b) If (3) is satisfied but (4) is violated, then reduce the total relay power *P*_*R*_ by a small constant Δ. (c) If (3) and (4) are violated, then reduce both the source and relay power *P*_*S*_ and *P*_*R*_ by a small constant Δ.

**Step 3 .**

Reallocate the source and the relay power equally on all the subchannels and find the optimal subchannel pairing by the Hungarian method; then go to Step 2.

The comparisons of the optimal algorithm and two suboptimal algorithms are summarized in Table 1. From Table 1, it is evident that both suboptimal algorithms do not fully exploit the potential of different channel conditions for network performance improvement since they carry out fixed subchannel pairing or equal power distribution on each subchannel that does not take into account different channel conditions, and thus they will result in sum rate reduction to some extent.

## 5. Performance Evaluation

### 5.1. Complexity Comparison

For the optimal algorithm (Algorithm 2), in each iteration it needs *N* ^2^ operations to compute ${\bar{P}}_{n,m}^{s}$ and ${\bar{P}}_{n,m}^{r}$, respectively. Then, it needs *N* ^2^ operations to calculate *U*_*n*,*m*_. The complexity of the Hungarian method is *O*(*N* ^3^). Hence, the total complexity of Algorithm 2 is *O*((*N* ^3^ + 3*N* ^2^)*E*_1_), where *E*_1_ is the iteration number of the subgradient method. It has been shown in [24] that *E*_1_ is a polynomial function with respect to the number of the dual variables that is four in Algorithm 2.

For suboptimal algorithm  1, it needs to compute ${\bar{P}}_{n,n}^{s}$ and ${\bar{P}}_{n,n}^{r}$ in each iteration; thus, it requires 2*N* operations. The subgradient algorithm needs *E*_1_ iterations to converge; hence its total complexity is *O*(2*NE*_1_).

The suboptimal algorithm  2 needs *E*_2_ iterations to calculate the power distribution and subchannel pairing, where *E*_2_ is related to the values of Δ, *T*_1_, *T*_2_, *P*_*S*_, and *P*_*R*_. It needs 2*N* operations to calculate the power distribution on each subchannel. The Hungarian method with complexity *O*(*N* ^3^) is used to calculate the subchannel pairing. Therefore, its overall complexity is *O*((2*N* + *N* ^3^)*E*_2_).

It should be noted that the computational complexity of the optimal algorithm is higher than the suboptimal algorithm  1. Although it is difficult to compare the value of *E*_1_ and *E*_2_, simulations in Section 5.2 reveal that suboptimal algorithm  2 has a lower complexity than suboptimal algorithm  1 in most scenarios.

### 5.2. Numerical Results

In this subsection, simulation results are presented to illustrate the performance of the proposed algorithms compared with the joint optimal algorithm in [8], which is indicated by optimal algorithm in [8] in the following figures and tables.

**Figure 2:**
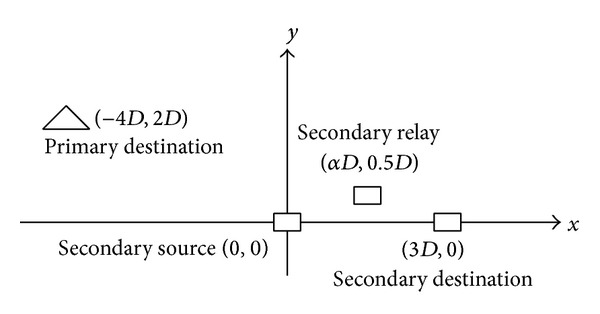
Simulation model.

Consider a simulation model shown in Figure 2, where the source and the destination of the SU are located at (0,0) and (3*D*, 0). The coordinate of the relay is (*αD*, 0.5*D*). The primary destination is located at (−4*D*, 2*D*). The total bandwidth is *B* = 1*M* Hz. The channel gain in any transmission pair contains a large-scale Rayleigh fading component and a large-scale path loss component with path loss factor 4. The small-scale fading is modeled as a frequency selective channel consisting of six independent Rayleigh multipaths. Each multipath component is modeled by Clarke's flat fading model. It is assumed that the power delay profile is exponentially decaying with *e* ^−2*l*^, where *l* is the multipath index. Therefore, the relative power of the six multipath components is [0, −8.69, −17.37, −26.06, −34.74, −43.43] dB [26]. The total powers at the source and the relay are assumed to be equal for simplicity. For the suboptimal algorithm  2, we set Δ = *P*_*S*_/100 = *P*_*R*_/100 in the following simulations. To show the performance of the algorithms in a fair and efficient manner, all the interested data in the simulations are averaged over 1000 independent channel realizations.

**Figure 3:**
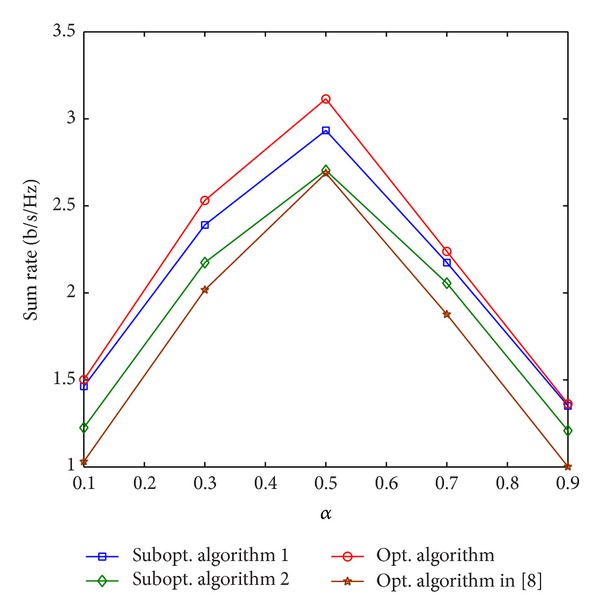
Sum rate versus the location of the relay.

First, simulations have been conducted to investigate the impact of the location of the relay on the sum rate. The parameters are set to be *D* = 150 m, *P*_*S*_ = *P*_*R*_ = 3 W, *T*_1_ = *T*_2_ = 0.15 W, and *N* = 14.  Figure 3 illustrates the sum rates of the four algorithms versus the location of the relay. As the increase of *α*, the distance between the source and the relay becomes longer, and the distance between the relay and the destination becomes shorter.  Figure 3 shows that all the algorithms reach the highest sum rate when the relay is located at the center between the source and the destination. The sum rate of the proposed optimal algorithm is higher than those of the other three algorithms under nearly all the values of *α*. When the relay is close to the source (*α* = 0.1) or the destination (*α* = 0.9), the gap between the sum rates of the proposed optimal algorithm and the proposed suboptimal algorithm  1 is very small, that is, less than 3%. The optimal algorithm in [8] always achieves the lowest sum rate compared with the proposed three algorithms in this paper. This is mainly because the optimal algorithm in [8] does not consider the direct transmission link while the proposed algorithms consider both the direct transmission link and the relay transmission link.

**Figure 4:**
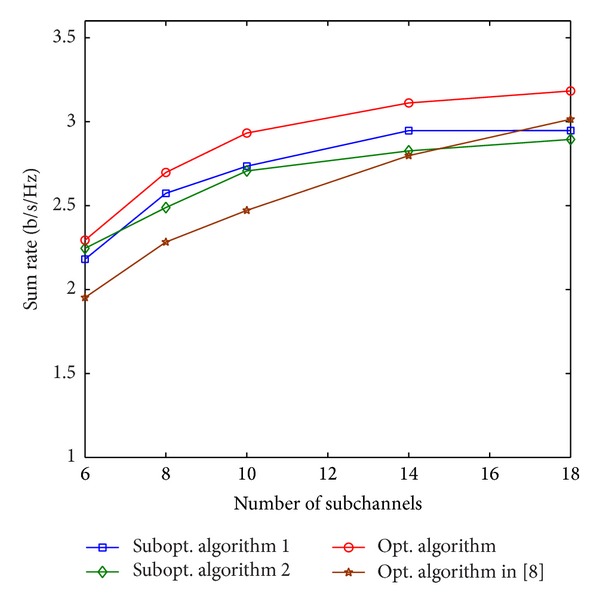
Sum rate versus the number of subchannels.

Figure 4 illustrates the sum rate comparison of the algorithms when the number of the subchannels changes. We set *D* = 150 m, *α* = 0.5, *P*_*S*_ = *P*_*R*_ = 3 W, and *T*_1_ = *T*_2_ = 0.15 W. As is evident from Figure 4, the sum rates of all the algorithms are increasing as the number of subchannels increases; that is because when the number of subchannels is large, there are big chances to allocate more power to the subchannel with higher channel gain, and thus, the total rate increases. Under different numbers of subchannels, the optimal algorithm always obtains a higher sum rate than the suboptimal algorithms and the optimal algorithm in [8], but the sum rates of the proposed two suboptimal algorithms are always very close to the optimal solutions; that is, the suboptimal algorithms  1 and 2 achieve more than 90% and 87% of the corresponding optimal solution, respectively. The sum rate of the optimal algorithm in [8] is always lower than that of the proposed optimal algorithm under different numbers of subchannels.

The computational complexity of the proposed algorithms is highly related to the number of subchannels as we analyzed in Section 5.1. To compare the computational complexity, we use Table 2 to show the average running time comparison of the algorithms when the number of subchannels changes. The simulation parameters of Table 2 have the same values as those in Figure 4. It is apparent from Table 2 that the proposed optimal algorithm and the optimal algorithm in [8] always need much longer average running time to obtain the final solution compared with the two suboptimal algorithms, but the running times of the proposed optimal algorithm and the optimal algorithm in [8] are also different. When the number of subchannels is 18, the running time of the optimal algorithm in [8] is nearly twice as much as that of the proposed optimal algorithm. Combined with the sum rate comparison in Figure 4, it is indicated that when the number of subchannels is larger than 10, the proposed optimal algorithm achieves a higher sum rate but with a much less running time compared with the optimal algorithm in [8]. In most cases, suboptimal algorithm  2 spends a little less time than suboptimal algorithm  1. From the simulation results of Figure 4 and Table 2, we conclude that the two proposed suboptimal algorithms can achieve near optimal solution with a significant average running time reduction.

**Figure 5:**
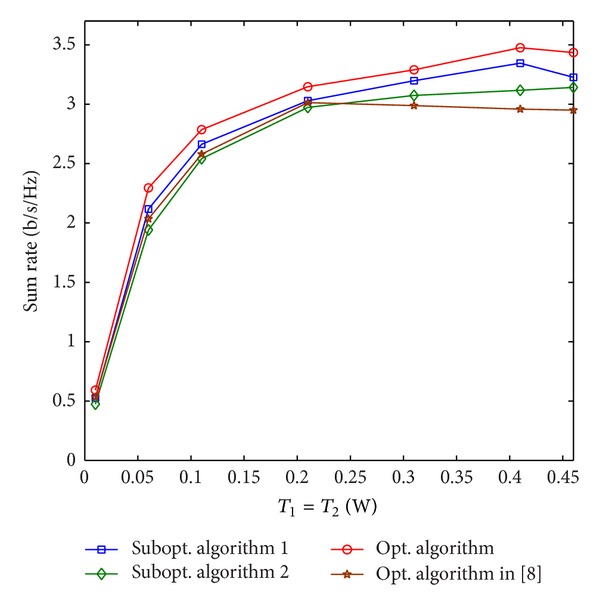
Sum rate versus *T*_1_.

Figure 5 shows the sum rate of the algorithms versus the interference power threshold *T*_1_(*T*_2_). The parameters are *D* = 150 m, *α* = 0.5, *P*_*S*_ = *P*_*R*_ = 3 W, and *N* = 14. It is apparent from Figure 5 that the sum rate is largely impacted by the value of *T*_1_(*T*_2_). When *T*_1_ is very small (*T*_1_ ≤ 0.1), the sum rates of the four algorithms increase dramatically as, the increase of *T*_1_, that is, because the interference power constraints are the dominating constraints in this case. As *T*_1_ increases, the total power constraints gradually become the dominating constraints, and thus, the sum rates keep changing in a small region. The proposed optimal algorithm achieves a higher sum rate compared with the other three algorithms under different values of *T*_1_. The ratios of the average sum rate achieved by suboptimal algorithms 1 and 2 to that achieved by the optimal algorithm are more than 88% and 80% under different values of *T*_1_, respectively.

**Figure 6:**
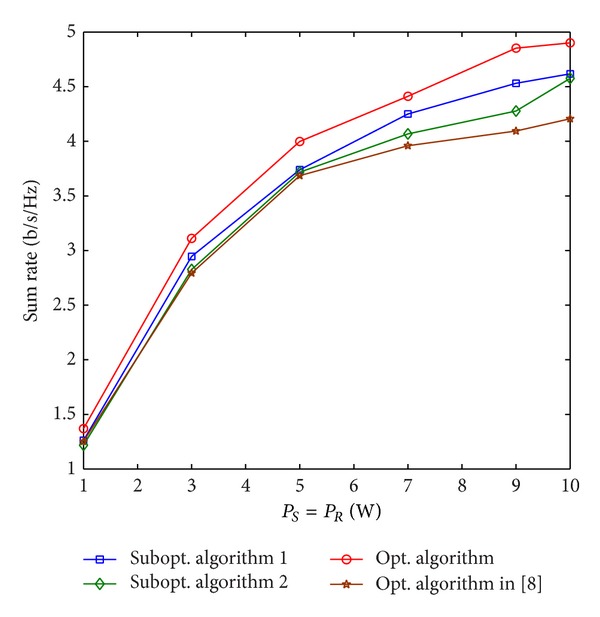
Sum rate versus the total power.

The sum rate versus the total power is illustrated in Figure 6. The parameters are set to be *D* = 150 m, *T*_1_ = *T*_2_ = 0.15 W, *α* = 0.5, and *N* = 14. It is seen from Figure 6 that the two suboptimal algorithms 1 and 2 achieve solutions that are more than 91% and 87% of the optimal solution for different values of *P*_*S*_, respectively. The sum rate of the optimal algorithm [8] is always nearly the same as the suboptimal algorithm  2 when *P*_*S*_ = *P*_*S*_ ≤ 5 W, and it becomes much smaller than that of suboptimal algorithm  2 when *P*_*S*_ becomes larger. The sum rate of all these algorithms increases as *P*_*S*_ increases. However, the sum rate increase rate gradually becomes slower as *P*_*S*_ increases. That is because, although *P*_*S*_ increases, the interference power constraints restrict the actual total power consumption on all subchannels.

## 6. Conclusion

In this work, we have considered the problem of subchannel pairing and power allocation in CRNs with the AF relay. The network model contains a PU and a secondary source-destination pair assisted by a secondary relay. The objective is to maximize the rate of the SU under the individual power constraints at the source and the relay and the subchannel pairing constraints. In order to protect the PU, the interference power constraints to the PU are also considered. The problem under consideration is formulated as a mixed integer programming problem. Via the dual decomposition method, we propose an algorithm that jointly optimizes the subchannel pairing and power allocation. To reduce the computational complexity, two suboptimal algorithms are proposed as well. The simulation result has shown that when the relay is located at the center between the source and the destination, the three proposed algorithms obtain the highest sum rate. It has also shown that the proposed suboptimal algorithms significantly reduce the average running time and achieve sum rates that are close to the optimal solution under different conditions. Therefore, the proposed suboptimal algorithms are very suitable for practical implementations where sum rate reduction can be tolerant to some extent. In the future, we will analyze the theoretical approximate ratio of the suboptimal algorithms to the optimal one and will investigate the performance of our proposed algorithms in the presence of imperfect channel state information.

## Figures and Tables

**Algorithm 1:**
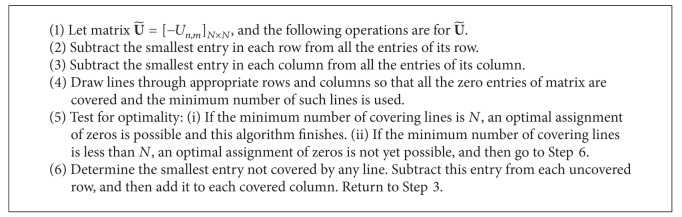
Hungarian method [27] for subchannel pairing.

**Algorithm 2:**
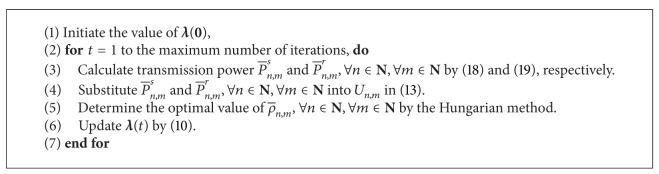
Optimal solution to (5).

**Algorithm 3:**
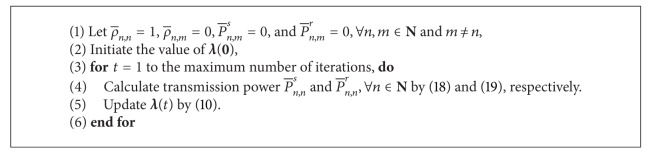
Suboptimal algorithm  1.

**Algorithm 4:**
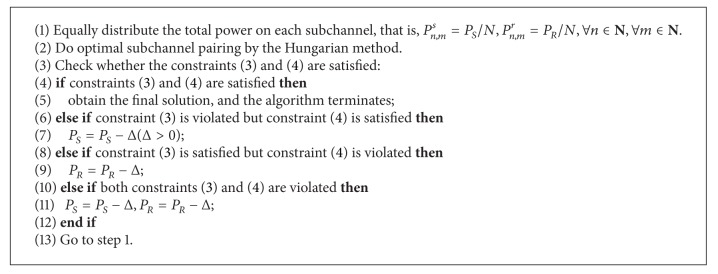
Suboptimal algorithm  2.

**Table 1:** Comparison of the proposed optimal algorithm and suboptimal algorithms.

Algorithm	Optimal algorithm	Suboptimal Algorithm 1	Suboptimal Algorithm 2
Resource allocation	Joint optimal subchannel pairing and power allocation	Given fixed subchannel pairing	Optimal subchannel pairing
		Optimal power allocation	Equal power distribution

**Table 2:** Average running time (in seconds) comparison for the four algorithms when the number of subchannels *N* changes, *D* = 150 m, α = 0.5,  *P*_*S*_ = *P*_*R*_ = 3 W, and *T*_1_ = *T*_2_ = 0.15 W.

*N*	Suboptimal algorithm 1	Suboptimal algorithm 2	Optimal algorithm	Optimal algorithm in [8]
6	0.4858	0.3908	1.6993	1.3974
8	0.5162	0.4072	2.6176	2.5733
10	0.5429	0.7631	3.8069	4.1873
14	0.6016	0.4782	7.0885	11.1492
18	0.6654	0.5678	12.1659	24.2083
